# Supplementary material for: Extended Invariant Information Clustering Is Effective for Leave-One-Site-Out Cross-Validation in Resting State Functional Connectivity Modeling
Source: Front Neuroinform. 2021 Dec 1;15:709179. doi: 10.3389/fninf.2021.709179 (PMC8671136; doi:10.3389/fninf.2021.709179)
Supplement: Supplementary file 1 [file Data_Sheet_1.PDF]

Supplementary Material

1 Supplementary Data

I. A comparison between the EIIC and another contrastive learning model

Our proposed EIIC method handles label pairs in the same way as the method proposed by Ktena et al. (2018). The latter is an example of a contrastive learning model, applied to the ABIDE dataset. Ktena et al. used a graph convolutional neural network, which reflected the information regarding brain structure in the RSFC data to train their model with a distance index. This helped to discriminate between AD and TC cases. In contrast, EIIC has the advantage that the output of paired learning is not a distance within a pair, but rather a model for clustering itself. Therefore, new incoming data can be directly labeled without making a pair with an existing one.

II. Mutual Information

The mutual information I of two random variables X and Y can be calculated using Equation (1) to evaluate the degree of dependence between the two random variables:

$$I(X, Y) = H(X) - H(X|Y)$$

$$= H(Y) - H(Y|X) \quad (1)$$

The mutual information refers to the difference between these two terms, which implies the extent to which knowing one of the two random variables can reduce the uncertainty about the other. The desired maximization of I(x,y) is achieved by minimizing and maximizing the conditional entropy H(X|Y) and marginal entropy H(X), respectively. The conditional entropy is minimized when X and Y are completely predictable from one to the other. Thus, it is optimal to assign data to one class with high probability. Considering that the marginal entropy is the uncertainty of X or Y itself, maximization avoids undesirable solutions that would assign data to a single class regardless of the label.

III. Weight updates

The definition of one "epoch" was somewhat unusual while training the EIIC model because of the alternation as an epoch unit that we first updated the weights for similar label pairs thoroughly, and then for different labels. The optimization method determines the procedure to update the weights based on the gradient of the loss function, calculated by an error back propagation. Moreover, Adam is a widely used method in neural network training.

The formula for the the update of the weight w is given below:

$$\begin{cases} m_t \leftarrow \beta_1 \cdot m_{t-1} + (1-\beta_1) \cdot g_t \\ v_t \leftarrow \beta_2 \cdot v_{t-1} + (1-\beta_2) \cdot g_t^2 \\ \grave{m}_t \leftarrow m_t/(1-\beta_1^t) \\ \grave{v}_t \leftarrow v_t/(1-\beta_2^t) \\ w_t \leftarrow w_{t-1} - \alpha \cdot \grave{m}_t/\left(\sqrt{\grave{v}_t} + \epsilon\right) \end{cases},$$

w_t denotes the weight updated t times and g_t is the gradient of the loss function for w_{t-1}. The learning coefficient α and those for the moving average of the gradients β1 and β2 are hyperparameters to be determined in advance. We set β1=0.9 and β2=0.999 for both models. We assigned the learning coefficient α, such that it oscillated every epoch as α_t according to the SGDR algorithm (Loshchilov and Hutterm, 2016), with a maximum value of α_{max}=0.0001.

$$\alpha_t = \frac{1}{2}\alpha_{max}\left(1 + cos\left(\frac{T_{cur}}{T_i}\pi\right)\right)$$

Initially, Tcur was set to 0 and T_i was set as a positive integer as a hyperparameter. With an increase in T_{cur} by 1 with each learning step, the learning coefficient α_t approaches 0. Upon reaching 0, T_i is updated and T_{cur} gets initialized to 0. The initial value of T_i was set to 2 and increased by 2 on each update. Through this operation, we could expect the learning coefficient to increase rapidly upon updating T_i. It assists the model in escaping from a local solution.

IV. Adjusting the mini-batch size

The tables highlight the results obtained with mini-batch sizes of 3,000 and 5,000.

V. Data and scripts

The data and the scripts are all available at: https://github.com/nokamot/EIIC

This is a repository of codes for An Extended Invariant Information Clustering, effective for the LOSO-CV in resting-state functional connectivity modeling.

a) Requirements

We used a docker container of pytorch/pytorch:1.8.1-cuda11.1-cudnn8-runtime for analysis. In addition, scikit-learn, pandas, and openpyxl are required.

b) How to run

1. Download data

Download the preprocessed ROI time series of the ABIDE dataset and phenotypic data from the download page of ABIDE Preprocessed.

2. Set config file

Prepare two directories to save the intermediate files (input data and labels) and final output (trained models).

Edit four relative path items of param_set.py as follows:

i) source_dir: Directory of intermediate files
ii) output_dir: Directory of result files
iii) preparation_params['label_file_path']: Phenotypic data

iv) preparation_params['path_structure']: Each ROI timeseries files

3. Train models and output results

Pull the Pytorch docker image.

sudo docker pull pytorch/pytorch:1.8.1-cuda11.1-cudnn8-runtime

Start the docker container mounting a directory, including these codes and necessary files, as mentioned in Section 2 (Set config file).

sudo docker run -it --rm --gpus device=0 -v /Path/to/codes/in/host:/Path/to/codes/in/container pytorch/pytorch:1.8.1-cuda11.1-cudnn8-runtime

Install the required packages by pip and run in the container.

pip install scikit-learn pandas openpyxl

cd /Path/to/codes/in/container

python run.py

2 Supplementary Table

Mini-batch size: 3,000

SITES	EIIC	SVM	RF	Heinsfeld et al.	Size	Age ± SD		
CALTECH	0.76	0.62	0.67	0.68	21	26.5	±	9.7
KKI	0.44	0.67	0.56	0.67	39	9.9	±	1.2
LEUVEN	0.62	0.64	0.59	0.65	61	18.1	±	5.0
MAX_MUN	0.48	0.55	0.48	0.68	42	27.9	±	11.1
NYU	0.63	0.68	0.62	0.66	169	15.2	±	6.5
OHSU	0.52	0.52	0.43	0.64	23	10.9	±	1.8
OLIN	0.72	0.68	0.60	0.64	25	17.1	±	3.4
PITT	0.64	0.69	0.53	0.66	45	19.2	±	6.8
SBL	0.76	0.56	0.52	0.66	25	35.2	±	8.6
SDSU	0.67	0.71	0.75	0.63	24	14.2	±	1.8

**STANFORD**	0.58	0.42	0.58	0.66	36	10.0 ± 1.6
**TRINITY**	0.63	0.74	0.51	0.65	43	17.2 ± 3.4
**UCLA**	0.74	0.65	0.56	0.66	72	13.3 ± 2.2
**UM**	0.71	0.71	0.63	0.64	112	14.5 ± 3.2
**USM**	0.75	0.79	0.46	0.64	61	23.7 ± 8.3
**YALE**	0.62	0.79	0.55	0.65	47	12.8 ± 2.9
**Mean**	0.64	0.65	0.56	0.65		

Mini-batch size: 5,000

SITES	EIIC	SVM	RF	Heinsfeld et al.	Size	Age ± SD
**CALTECH**	0.62	0.62	0.67	0.68	21	26.5 ± 9.7
**KKI**	0.69	0.67	0.56	0.67	39	9.9 ± 1.2
**LEUVEN**	0.59	0.64	0.59	0.65	61	18.1 ± 5.0
**MAX_MUN**	0.60	0.55	0.48	0.68	42	27.9 ± 11.1
**NYU**	0.64	0.68	0.62	0.66	169	15.2 ± 6.5
**OHSU**	0.43	0.52	0.43	0.64	23	10.9 ± 1.8
**OLIN**	0.72	0.68	0.60	0.64	25	17.1 ± 3.4
**PITT**	0.51	0.69	0.53	0.66	45	19.2 ± 6.8
**SBL**	0.56	0.56	0.52	0.66	25	35.2 ± 8.6
**SDSU**	0.67	0.71	0.75	0.63	24	14.2 ± 1.8
**STANFORD**	0.56	0.42	0.58	0.66	36	10.0 ± 1.6
**TRINITY**	0.58	0.74	0.51	0.65	43	17.2 ± 3.4
**UCLA**	0.58	0.65	0.56	0.66	72	13.3 ± 2.2
**UM**	0.67	0.71	0.63	0.64	112	14.5 ± 3.2
**USM**	0.75	0.79	0.46	0.64	61	23.7 ± 8.3
**YALE**	0.60	0.79	0.55	0.65	47	12.8 ± 2.9
**Mean**	0.61	0.65	0.56	0.65		

3 Supplementary Figures

A

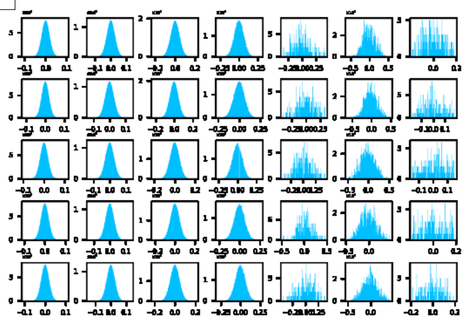

B

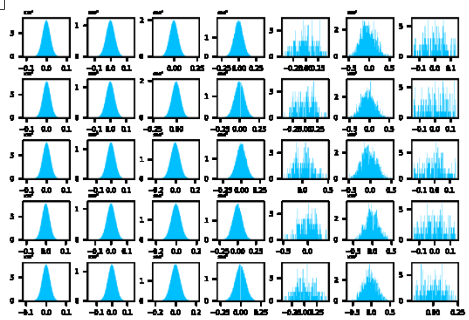

C

D

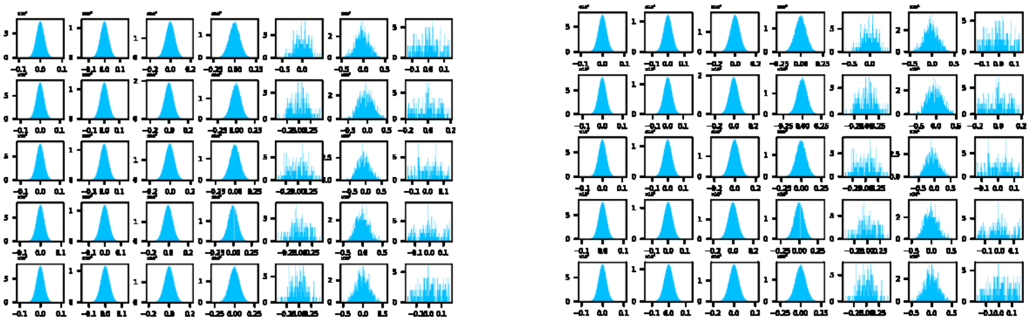

Four typical examples of the site-wise histograms of the fully connected layers' weights in the EIIC computation with the number of bins set to 100. These are A:SBL(0.84), B:CALTECH(0.81), C:OHSU(0.48), and D:STANFORD(0.47), which are four organizations providing source data recording the two highest and two lowest accuracy rates. All the histograms for the 16 organizations in total (“hist_300dpi.zip”) and the Python code for computation (“check_weights.ipynb”) are published and available at: https://github.com/nokamot/EIIC . The numerical data for the overall layers’ weights will be provided upon the request of readers under the form of “result_models_bs500_<site>.bf”, of which the file size is approximately 800 Mb. In each histogram, the five rows correspond to each fold of the 5-fold cross-validation, whereas the seven columns correspond to each layer in the way that they lined up in the progress order from the left to the right. The leftmost four columns in each histogram represent the layers in the contrastive prior learning (simple IIC structure) for the first step (Figure 1), of which the weights are fixed and inherited to the transfer learning for the second step. These histograms for the fully connected layers took a sort of bell shape similar to the normal distribution around 0, regardless of the resulted classification accuracy. Conversely, the rightmost three columns in each histogram stand for the output layers, including IIC head, IIC_oc head (Figure1 A for the prior learning), and Classifier_head (Figure1 B for the posterior learning), respectively. When comparing the weight distributions of the IIC head (third from the right) and the Classifier head (the far right) sharing the same number of output nodes, it turned out that for the majority of the data source sites, the standard deviation of the weights at the latter was reduced to almost half of that at the former, signifying that the final discrimination in the posterior learning was based on the whole output from the proximate layer without reflecting the influence of some particular nodes there.

4 References

Ktena, S.I., Parisot, S., Ferrante, E., Rajchl, M., Lee, M., Glocker, B., Rueckert, D. (2018) Metric learning with spectral graph convolutions on brain connectivity networks. NeuroImage 169, 431-442.

Loshchilov, I., and Hutterm F. (2016). Sgdr: Stochastic gradient descent with warm restarts. arXiv Preprint. arXiv:1608.03983.
